# How Dietary Choices and Nutritional Knowledge Relate to Eating Disorders and Body Esteem of Medical Students? A Single-Center Cross-Sectional Study

**DOI:** 10.3390/nu16101414

**Published:** 2024-05-08

**Authors:** Aureliusz Andrzej Kosendiak, Bartosz Bogusz Adamczak, Zofia Kuźnik, Szymon Makles

**Affiliations:** 1 Department of Physical Education and Sport, Wroclaw Medical University, 51-601 Wroclaw, Poland; 2 Student Scientific Association, Department of Physical Education and Sport, Wroclaw Medical University, 51-601 Wroclaw, Poland

**Keywords:** students, students’ knowledge, medical university education, diet, feeding behavior, caloric restriction, feeding and eating disorders, body image, attitude to health, young adult

## Abstract

Eating disorders and body image concerns are increasingly prevalent issues among young individuals, with medical students being particularly vulnerable due to heightened stress levels. This study enrolled 879 medical students to investigate these concerns. The KomPAN questionnaire was utilized to assess dietary habits and knowledge, the Body Esteem Scale (BES) to evaluate body satisfaction, and The Eating Attitudes Test (EAT-26) to identify eating disorders. A higher level of nutritional knowledge was found to be statistically significantly associated with attempts at excessive calorie restriction among women (β = 0.0864) and negatively among men (β = −0.2039). Moreover, it was negatively associated with self-control of food intake only among men (β = −0.2060). Furthermore, a higher BMI was associated with attempts of excessive calorie restriction in both women and men (β = 0.1052 and β = 0.1656, respectively) and negatively with self-control of food intake (β = −0.0813 and β = −0.1453, respectively). A higher BMI was associated with poorer body esteem across all variables in both genders, except for upper body strength among men. Nutritional knowledge did not correspond with any of these variables, while dietary quality was positively associated with physical condition in women and with physical condition, physical attractiveness, and upper body strength in men. Our study findings suggest that dietary interventions could be improved by considering gender-based behavioral differences and focusing on portion control for individuals with a higher BMI. Caution is warranted in extrapolating the results to the general population due to the specific nature of the study population.

## 1. Introduction

In recent years, there has been a concerning rise in the prevalence of eating disorders (EDs) among teenagers and young adults [1]. A 2015 systematic review found that Internet and social media platforms contain appearance-related content promoting extreme body shapes or behaviors [2]. The review established a consistent relationship between Internet use, especially appearance-focused social media, and heightened body image and eating concerns. Adolescents, in particular, are vulnerable to these effects due to their developmental characteristics. This trend may be attributed, in part, to the pervasive influence of unrealistic beauty standards perpetuated by social media platforms and the accompanying pressure from peers to conform to these ideals from an increasingly young age [3]. The ubiquitous nature of social media facilitates constant exposure to curated images portraying unattainable body standards, fostering a culture of comparison and dissatisfaction with one’s own appearance [4]. Furthermore, the normalization of extreme dieting practices and the glorification of thinness within online communities can contribute to the development of disordered eating behaviors [5]. Additionally, a 2018 meta-analysis found that body checking and body image avoidance lead to EDs  [6]. In a 2023 study, participants assigned to watch videos related to diet culture on a social media platform demonstrated greater escalation in dietary restriction and an inclination toward exercise, alongside diminished improvements in positive mood, compared to those exposed to neutral content [7]. Notably, videos promoting diet culture had a more detrimental impact on body image and eating concerns when contrasted with anti-diet content. Peer pressure exacerbates these influences, as individuals strive to meet perceived expectations of attractiveness and acceptance within their social circles [8]. Furthermore, among adolescents, there is a notable positive correlation between peer pressure and heightened anxiety levels, which can be a risk factor of developing EDs [9].

The selected cohort for our investigation comprises medical students, recognized as a demographic encountering manifold challenges and stressors with heightened levels of anxiety and stress relative to the general population. The medical school experience is characterized by rigorous academic demands, comprising intricate coursework, rigorous study schedules, and unconventional academic calendars [10]. Other prevalent stressors among medical students delineated in the research may include elevated parental expectations, frequent examinations, and apprehensions regarding future prospects [11]. According to a study conducted in 2016, medical students exhibit almost 4 times higher prevalence rates of major depression in contrast to the general population [12]. This elevated occurrence of mental disorders among newly enrolled medical students compared to the general populace may be attributed to the selection of particularly vulnerable personalities, such as high performers, and the stress induced by the highly competitive environment stemming from stringent entry requirements. It has been postulated that medical students possess certain personality traits rendering them susceptible to mental distress [13,14]. A study found that within the medical student cohort, unhealthy dietary practices, such as breakfast omission and irregular meal schedules, were notably associated with heightened stress levels. Additionally, stress-induced eating exhibited a pronounced prevalence, particularly among female medical students [15].

Furthermore, another contributing factor to the precarious mental well-being of medical students is their suboptimal sleep quality. A 2019 study conducted among medical students identified poor sleep quality as an independent risk factor for a heightened propensity toward EDs, observed consistently across both male and female participants [16]. Empirical evidence suggests that sleep quality significantly influences mental health, with the majority of students reporting poor sleep quality [17]. A study conducted in 2020 revealed preliminary evidence suggesting that disrupted sleep may play a particularly significant role in the onset and progression of EDs, commencing in adolescence [18].

Notably, research shows medical students constitute a demographic with a heightened vulnerability to EDs. A 2018 meta-analysis revealed that medical students have an overall pooled prevalence rate of ED risk at 10.4% [19], while a 2021 meta-analysis found that the lifetime prevalence of EDs in Western countries was 1.89%, with females having a higher rate of 2.58% [20]. According to the aforementioned study [19], medical students are at high risk of developing mental health problems, including EDs, due to factors such as academic stress, heavy workloads, and exposure to illness and death during their education. Moreover, in a 2018 study on medical students, the prevalence rates of social anxiety disorder (SAD) symptoms and body image disturbance (BID) were 36.3% and 34.7%, respectively; depressive symptoms occurred in 8.8% of the sample. Furthermore, SAD symptoms were independently associated with BID [21]. The stigma surrounding mental health disorders and EDs can lead to denial, self-medication, and delayed treatment, exacerbating the severity and persistence of EDs [19].

Exploring the risks of EDs and body image disorders within the context of medical students holds significance, given their exposure to nutritional science education throughout their academic training. Nonetheless, assessing the comprehensiveness of this education proves challenging due to inconsistencies in research findings. A 2021 study demonstrated that medical students achieved a significantly elevated mean score on the Nutritional Knowledge Questionnaire compared to a control group, displaying nearly one-third higher scores [22]. Moreover, a separate study focusing on UK students demonstrated that those pursuing healthcare-related disciplines exhibited higher median knowledge scores compared to their non-healthcare counterparts [23]. Although, findings from a 2019 study conducted in the Netherlands indicated inadequate nutritional knowledge among medical students [24]. Furthermore, a systematic review of the literature underscored the prevalence of poor nutritional knowledge among medical students across various domains of nutrition, encompassing both fundamental and specialized areas [25]. While medical students typically possess greater nutritional knowledge compared to the general population, their level of understanding remains insufficient relative to the expected expertise for physicians. Understanding the interplay between nutritional knowledge and dietary behaviors among medical students holds implications of significance, because of their role as future healthcare professionals.

The primary objective of this study is to analyze how body self-esteem and the risk of EDs are associated with the dietary knowledge, diet quality, and BMI of medical students. We have chosen these variables because they are easily measurable and can also be modified in a relatively realistic manner. Our hypotheses posit that individuals exhibiting lower levels of body esteem and heightened risk of EDs will demonstrate associations with abnormal BMI levels. Furthermore, it is anticipated that those with a diminished risk of eating disorders and elevated levels of self-body esteem will exhibit greater dietary knowledge. Additionally, we hypothesize that individuals with heightened levels of self-body esteem will be characterized by superior dietary quality. This investigation holds significance within this specific cohort, given their prospective roles in medical professions, which demand a heightened resilience to stressors. Additionally, the anticipation for these individuals to advocate for healthier lifestyles among their patients, alongside their social responsibilities, accentuates the necessity of examining their own behaviors and coping mechanisms. Consequently, it is imperative to closely monitor the diverse habits and coping strategies employed by students pursuing degrees in medical-related disciplines.

## 2. Materials and Methods

### 2.1. Study Design and Participants

This research was designed as a cross-sectional study involving first-year students at Wroclaw Medical University. The primary objective was to explore the relationship between eating disorders and body perception and students’ dietary behaviors and knowledge.

Data collection took place in October 2023. During this phase, students attending mandatory physical education classes were provided with links to completely anonymous surveys. To ensure comprehensive representation, invitations were extended to all students who potentially met the inclusion criteria, aiming to capture diverse perspectives within the cohort.

A crucial aspect of this study’s design was the meticulous adherence to ethical principles. The participants were fully informed about the study’s purpose, and their voluntary participation was sought through a process of informed consent.

**Figure 1:**
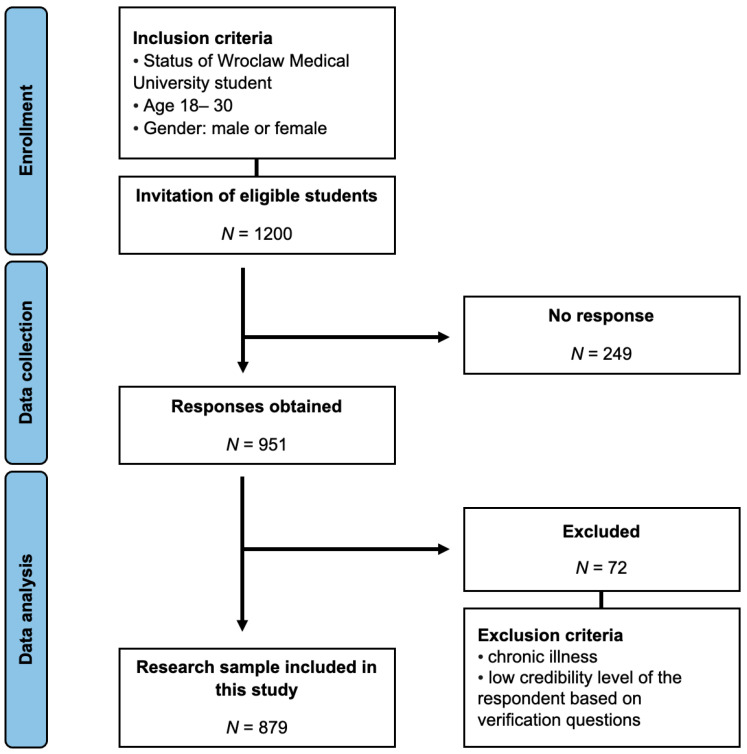
Study selection process.

For a comprehensive understanding of the data collection process and the inclusion and exclusion criteria, readers are directed to Figure 1.

### 2.2. KomPAN

The KomPAN [26] is a comprehensive survey tool developed by the Polish Academy of Sciences. It is used as a robust instrument for the evaluation of dietary habits and nutrition beliefs in individuals.

The initial segment focuses on the frequency of consumption of various food types, encompassing 33 questions. Among these, 9 questions serve as distractors, while 24 questions contribute to the calculation of two scales: the Non-Healthy Diet Index (nHDI-14) and the Pro-Healthy Diet Index (pHDI-10). These scales evaluate the frequency and types of consumed food, with the nHDI-14 encompassing 14 food groups potentially detrimental to health and the pHDI-10 comprising 10 food groups with potential health benefits. The intensity of dietary characteristics is determined by the scores achieved on each scale, categorized as low (0–33%), medium (34–66%), or high (67–100%). Additionally, to enable statistical analysis, we introduced a new parameter termed the Dietary Score (DtSc). This metric was computed by subtracting the percentage score of the nHDI-14 from that of the pHDI-10. The resulting range of values spans from −100 to 100, offering insight into the dominant dietary pattern of each participant. The DtSc is constructed upon existing validated parameters, utilized solely for facilitating analysis and demonstrating the prevailing trend among variables.

The subsequent segment of the questionnaire consists of a single-choice nutrition beliefs test, encompassing 25 questions meticulously designed to probe the respondent’s comprehension of food and nutrition. Respondents are presented with True/False/I’m not sure answer options, with one point allocated for a correct response, while zero points are awarded for an incorrect or uncertain reply. The aggregate score from this section is denoted as dietary knowledge (DK).

To fortify the questionnaire’s integrity, three sets of validation questions are strategically embedded to authenticate respondent reliability and facilitate the exclusion of untrustworthy responses from subsequent analyses.

It is noteworthy that the KomPAN questionnaire on nutrition has undergone a rigorous validation process, emerging as a dependable and valuable tool for researchers delving into the intricate realms of dietary habits and nutrition beliefs among individuals [27,28].

### 2.3. Body Esteem Scale (BES)

The Polish version of the BES [29,30] was utilized to determine participants’ perceptions of their own bodies. The scale consists of 35 body parts and associated characteristics, for which participants must assess their own feelings on a scale from 1 (strongly negative feelings) to 5 (strongly positive feelings). Three subscales are calculated from the total score, separately for women and men, which cannot be directly compared due to variations in the included questions.

For women, these subscales comprise the following: sexual attractiveness (SA), concerning the perception of physical features unalterable through exercise and linked to sexuality; weight control (WC), encompassing body parts amenable to change through physical activity or dietary modifications; and physical condition (PC), referencing aspects such as strength and endurance.

For men, the subscales consist of the following: physical attractiveness (PA), related to traits defining a man as handsome and masculine; upper body strength (UB), based on the appearance and strength of the upper body; and physical condition (PC), encompassing strength, endurance, and agility.

The BES is a questionnaire with established validity and reliability, demonstrated through research, including studies conducted within the Polish population [30].

### 2.4. The Eating Attitudes Test (EAT-26)

The EAT-26 is a questionnaire utilized for assessing psychological features associated with eating disorders [31]. It comprises 26 questions addressing feelings and behaviors related to eating. Each question is rated on a scale from 1 (never) to 6 (always)—with higher scores indicating greater severity of the disorder. The questionnaire can be subdivided into three subscales: the dieting (DI) factor, characterized by vigilant monitoring of calorie, carbohydrate, and sugar intake driven by a desire to achieve a thinner body; the bulimia and food preoccupation (B&FP) factor, encompassing behaviors such as purging after meals and excessive preoccupation with food; and the oral control (OC) factor, reflecting tendencies toward self-control of food intake and portion control.

The EAT-26 is a questionnaire with established validity and reliability, demonstrated through research, including studies conducted within the Polish population [32,33].

### 2.5. Statistical Analysis

In this study, Microsoft Excel version 16.77 (Redmond, WA, USA) was utilized for data cleansing, curation, and computation. Statistical analyses, including the identification of significant relationships, were conducted using Statistica 13 (Statsoft, Kraków, Poland).

The collected survey data exhibited a non-normally distributed pattern, as determined by the Shapiro–Wilk test. The descriptive analysis encompassed the frequency and percentage measures for qualitative variables and the mean, median, and interquartile range (IQR) for quantitative variables. The Mann–Whitney U test was employed for comparing two quantitative variables. All the analyses were conducted at a significance level of *p* < 0.05.

Following the verification of assumptions, two separate linear regression models were constructed for each gender, considering variables from the BES and EAT-26 questionnaires. Model 1 incorporated DK and the DtSC from the KomPAN questionnaire, while Model 2 additionally included BMI. Standardized coefficients (β) with accompanying 95% confidence intervals (95% CI) were presented, alongside the percentage of variance explained (R^2^) and the F-value. Variables that reached statistical significance were emphasized in bold to improve the clarity of the tables.

## 3. Results

The general characteristics of the study participants are presented in Table 1. A total of 879 individuals took part in this study, with the majority, comprising 75.1%, being women. The largest proportion of participants resided in major urban centers (43.9%), while nearly a quarter lived in rural areas, and the remainder in small to medium-sized towns. A majority of the study population fell within the normal BMI range, accounting for 77.3%, whereas groups classified as overweight or obese and underweight were roughly equally represented, slightly exceeding 11% each.

Table 2 presents the values and differences in the variables under investigation. It is noteworthy that there was a significant difference between genders in terms of the BMI levels (higher in men) and Dietary Score (higher in women). However, there was no significant difference observed in dietary knowledge or eating disorders between the genders.

Table 3 presents two linear regression models concerning the studied women—Model 1, which includes dietary knowledge and the Dietary Score, and Model 2, which additionally incorporates BMI. Nutritional knowledge among women influences only the level of dieting, indicating that a higher level of nutritional knowledge is positively associated with a greater tendency toward restricting calories. Regarding diet quality, represented by the Dietary Score, there is a positive association with dieting and physical condition. Concerning BMI, there is a negative association with each of the variables related to body self-esteem. Furthermore, the observed positive association with the dieting variable and negative association with oral control suggest that women with a higher BMI are more concerned with the higher calorie intake rather than the quantity of food consumed.

Table 4 presents two linear regression models concerning the studied men—Model 1, which includes dietary knowledge and the Dietary Score, and Model 2, which additionally incorporates BMI. Attention is drawn to the negative relationship between dietary knowledge and variables concerning eating disorders (DI, B&FP, and OC), suggesting that higher nutritional knowledge reduces psychological features associated with eating disorders. Importantly, such a relationship did not occur among women; furthermore, it was the opposite in the DI variable. Diet quality was positively associated with the dieting variable, as well as physical attractiveness and upper body strength concerning self-body evaluation. Regarding BMI, similar to women, there was a positive relationship with dieting and a negative one with oral control. Regarding body self-esteem, higher levels of BMI corresponded with lower levels of the physical attractiveness and physical condition variables, although there was no such change in the upper body strength variable.

## 4. Discussion

The objective of our study was to investigate how self-body esteem and the risk of EDs are associated with the nutritional habits and knowledge of medical students attending Wroclaw Medical University. Our study revealed that among women, a higher level of potentially excessive calorie restriction (DI), one of the predictors for EDs, was associated with higher levels of nutritional knowledge. It is noteworthy that individuals with EDs tend to have a deeper understanding of nutrient sources than the general population [28]. Conversely, among men, lower ED risk determinants, such as DI, B&FP, and OC, were associated with greater nutritional knowledge, which aligns with our hypothesis. These gender disparities may be attributed to several factors. Firstly, while men can also experience body dissatisfaction, women tend to experience more severe and prevalent dissatisfaction, often intertwined with their sense of self-worth [34]. Additionally, societal influences, including dieting culture, play a role, with women typically pressured toward achieving thinness, while men are encouraged to attain a muscular physique indicative of health [35]. Research has demonstrated that a higher energy intake coupled with progressive resistance training leads to more significant increases in hypertrophy compared to situations involving lower caloric conditions. This observed trend may elucidate our study’s findings regarding the lower prevalence of ED risk factors among men with greater nutritional knowledge, potentially associated with a preference for a muscular physique over a slender one [36]. Additionally, experts assert that dieting and the pursuit of thinness are more prevalent among females than males [37]. Furthermore, although more men are overweight, a greater number of women report dissatisfaction with their weight. It is associated with the research showing that women experience diet culture more than men and are more likely to engage in ‘corrective’ measures such as dieting and to develop ED [38]. This underscores the importance of tailoring nutritional education interventions to address gender-specific needs. Specifically, efforts to enhance women’s nutritional knowledge should prioritize promoting healthy habits while recognizing and mitigating the potential negative impact of societal pressures and discriminatory practices. Such nuanced approaches are important for ensuring the effectiveness and appropriateness of nutritional education initiatives across genders.

In our study, both male and female participants exhibited an intriguing trend, partially in line with the initial hypothesis: individuals with a higher BMI tended to have a higher DI score, while their OC was lower. This suggests that overweight individuals in our cohort tended to prioritize calorie restriction over monitoring the volume and quantity of food consumed. Such an association may potentially contribute to their weight gain. The current literature provides limited research evidence of this pattern. Across numerous studies, calorie restriction has been consistently linked to a reduction in energy expenditure exceeding what can be accounted for by losses in metabolic mass, including fat-free mass and fat mass [39]. However, without careful consideration of food quantity and a lack of control over eating, calorie restriction may prove ineffective and potentially lead to binge eating and the onset of an ED [40]. One potential interpretation of our results may be linked to the inclination of overweight individuals to opt for commonly available, lower calorie food and beverage options, often featuring sugar-free variants. However, separating sweetness from caloric content partially stimulates food reward pathways, potentially heightening appetite. Moreover, a diminished reward response may play a role in contributing to obesity [41]. This phenomenon could explain lower OC, higher overall food consumption, and an elevated BMI in our study. Existing studies demonstrate this trend wherein a clinically significant connection is observed between extreme weight-loss dieting and the onset of binge eating, particularly in the development of bulimia nervosa [41]. Moreover, a 2016 meta-analysis reveals that binge eating or loss of control overeating was prevalent among more than one quarter of children and adolescents diagnosed with overweight and obesity [42]. This finding underscores the importance of interventions focusing not only on counting calories but also on addressing portion sizes and overall dietary habits. By emphasizing the importance of mindful eating and portion control, interventions can potentially assist individuals in achieving and maintaining a healthier weight.

The results of our study indicate a positive association between better dietary quality and more instances of potentially excessive calorie restriction, alongside higher scores in self-perceived physical condition among both genders, with the latter corresponding with our initial hypothesis. These findings are consistent with prior research, which indicates that superior overall diet quality correlates with objective assessments of physical performance, for example, a greater number of pull-ups and push-ups [43]. Moreover, this corresponds with our observations within the male cohort, indicating that improved diet quality is associated with the UB, consequently resulting in higher scores for PA. This trend is not applicable to women, potentially due to the aforementioned influence of the prevailing diet culture, which places greater pressure on them, and a higher proportion of women expressing discontent with their weight compared to men.

Additionally, the outcomes of our study suggest a positive association between better dietary quality and more instances of potentially excessive calorie restriction for both men and women. However, we have found no existing research acknowledging this phenomenon. Various factors may contribute to these observed results. One of the possible explanations is that individuals demonstrating an elevated commitment to enhancing dietary quality may also display a greater inclination toward strict adherence to dietary guidelines, consequently leading to an inclination toward calorie restriction. Subsequently, societal norms and pressures pertaining to dieting and body image may exert influence, prompting individuals to embrace more restrictive eating practices in pursuit of perceived health and aesthetic ideals.

In our study, we observed a significant association between lower levels of body self-esteem and a higher BMI across both male and female participants. Specifically, variables such as SA, WC, PC, and PA were negatively associated with a higher BMI, which aligns with the initial hypothesis. These findings are in line with the existing research, including a 2016 study focusing on female university students, which revealed a significant and consistent correlation between BMI and body dissatisfaction (BD) [44]. Moreover, BMI values were identified as useful predictors of BD risk among young females. Similarly, research has shown that men with higher BMIs, classified as overweight or obese, tend to exhibit lower rates of body satisfaction [45]. It is plausible that the association between lower body self-esteem and being overweight or obese may be influenced by societal pressures and unrealistic beauty standards perpetuated by various media outlets, including social media, the fashion industry, and the film industry [46].

A 2021 study demonstrated a positive correlation between social media usage and decreased body satisfaction and overall well-being [47]. The relationship between social media use and well-being can be attributed to mechanisms such as social and appearance comparisons. Research shows that mass media influence correlates with heightened BD among adolescents, which, in turn, elevates the risk of developing an ED in both genders, particularly in girls [48]. Additionally, both mass media influence and BMI predict BD, which subsequently predicts ED risk in both girls and boys. The singular variable associated with male body self-esteem that remained constant despite a higher BMI was upper body strength. While the existing research generally indicates a decrease in muscular strength with a higher BMI [49], our study yielded a different outcome. This discrepancy may stem from the limitation of the BMI as a measurement tool, as it does not distinguish between fat and fat-free mass, such as muscles and bones. Consequently, certain individuals may have been misclassified as overweight when, in reality, they exhibited an athletic physique with an appropriate weight [50].

During the assessment of BMI, we noted a prevalence of participants classified as either overweight (BMI < 25) or underweight (BMI > 18.5) at 11% each. Sixteen participants in the study (1.82%) were categorized as obese, defined by a BMI exceeding 30. According to a study conducted in 2015, the incidence of obesity among non-medical Polish university students stood at 3.9% [51]. A previous longitudinal study conducted on Polish medical students from 2019 to 2021 [52], and another in 2023 [17], similarly reported an average of one-quarter of students with an abnormal BMI. Our study exhibited a favorable outcome in comparison to various investigations across populations, including those in Poland (36.3%) [53] and Spain (30.3%) [54]. Conversely, an earlier study in 2022, focusing on a comparable but smaller sample of medical students from Wroclaw, disclosed a notably lower abnormal BMI rate of 17.5%, suggesting a more positive outcome [55]. Hence, it is advised to acknowledge that the presence of either underweight or overweight students constitutes a concern.

Additionally, our findings revealed notable disparities between genders in various aspects of nutritional behavior. An analysis of Table 2 helped to determine whether discrepancies in regression outcomes between genders result from differential responses to identical conditions or from inherent gender-based differences in the conditions themselves. Interestingly, despite these differences, both male and female participants exhibited comparable levels of nutritional knowledge, suggesting a shared understanding of dietary principles among the study cohort. This trend corresponds with findings from a 2023 study conducted among university students in Saudi Arabia. Despite a marginally higher level of nutritional knowledge observed among females, the scores for nutritional knowledge were closely comparable between genders [56]. Furthermore, in a 2011 study, dietitians showed superior nutrition knowledge compared to all healthcare professionals, except medical doctors. They surpassed both patients and controls in all aspects of nutrition. However, other healthcare professionals had similar knowledge to patients [57]. Notably, in our study, women displayed a significantly higher quality of diet represented by the DtSc, while men exhibited a higher BMI. This observation aligns with broader research trends within the general population, where men are more frequently overweight than women, which can be often attributed to differences in dietary quality [58]. A study involving medical students from Poland, Belarus, Lithuania, and Russia revealed that dietary choices significantly influenced their BMI and weight. Specifically, increased consumption of pork fat, lard, noodles, sweet drinks, light bread, and butter was notably associated with higher BMI values, with pork fat and lard exerting the most pronounced influence, followed by noodles and sweet drinks [59]. A 2015 systematic review found that in general, women tend to have better diets than men on average and older adults generally exhibit superior dietary habits compared to younger adults [60].

As for the future implications and perspectives, educational initiatives targeting the general public should prioritize promoting balanced dietary habits and positive body image perceptions, particularly addressing the disproportionate impact of societal pressures on women. Similarly, interventions within medical education programs are important for equipping future healthcare professionals, focusing on understanding and addressing the complex interplay between nutrition, body image, and mental health. It is advised to recognize that medical education primarily emphasizes patient care, with self-care often being overlooked. Interventions to address psychological challenges among medical students must thus be sensitive to the unique stresses they face. Furthermore, while the expectation for doctors to serve as exemplars of healthy lifestyles and behaviors persists, it may be unrealistic given the various demands of their profession. Support mechanisms should be multifaceted, integrating tailored resources and proactive mental health initiatives within medical culture. In 2019, a study determined that psychotherapeutic interventions for bulimia nervosa resulted in notably enhanced post-treatment levels of self-esteem compared to control conditions. Similarly, psychotherapy administered for binge eating disorder demonstrated significant post-treatment enhancements in self-esteem compared to control groups [61]. A meta-analysis conducted in 2014 revealed that psychotherapeutic modalities encompassing cognitive, behavioral, and mindfulness approaches were linked to reductions in anxiety symptoms among medical students. Additionally, interventions integrating psychoeducation, interpersonal communication strategies, and mindfulness meditation were associated with diminished burnout among both physicians and medical students [62]. Psychotherapeutic interventions hold promise in enhancing self-esteem and mitigating symptoms of anxiety and burnout among medical students, indicating the potential efficacy of psychotherapy in preventing and addressing eating disorders. By fostering a supportive environment and providing accessible resources, healthcare systems can bolster the well-being of medical professionals, ensuring the sustained delivery of high-quality patient care.

It is relevant to recognize the divergent effects of dietary knowledge on women and men. While men typically adopt healthier habits in response to better dietary knowledge, women tend to engage in potentially excessive calorie restriction. Hence, educational programs should be tailored differently based on their target audience’s gender-specific responses. Further research should explore the underlying mechanisms driving gender differences in dietary habits and body image perceptions to inform targeted intervention strategies. Additionally, when educating individuals on healthy eating habits, it is crucial to underscore the potential consequences of an excessive calorie deficit, including the development of binge eating and loss of oral control, which can predispose individuals to EDs. As such, these concerns should be adequately addressed within interventions and educational programs. Furthermore, in forthcoming weight loss interventions and educational initiatives, emphasis should be placed on prioritizing the restriction of portion sizes and overall food quantity, rather than exclusively concentrating on calorie reduction. This is imperative as overweight patients may lean toward integrating lower calorie foods into their diet without regulating the overall daily quantity, potentially leading to counterproductive outcomes.

While this study provides valuable insights, it is necessary to acknowledge its inherent limitations. The research cohort, composed solely of students from Wroclaw Medical University, represents a specific demographic subset, thereby constraining the generalizability of the findings to a broader societal context. Furthermore, the utilization of an online survey methodology introduces the potential for participant misinterpretation of questions, despite researchers’ efforts to provide clear instructions. Moreover, questionnaire-based studies solely rely on self-reported declarations. Moving forward, our intention is to conduct comprehensive body composition assessments alongside questionnaire-based evaluations, thereby yielding a more objective portrayal of the subject matter. Consequently, caution should be exercised when extrapolating the observed behavioral characteristics to the broader adult population. It is recommended that future research endeavors encompass a more diverse and extensive participant pool to enhance the applicability and depth of the findings delineated in this paper.

## 5. Conclusions

The primary objective of this study is to analyze how body self-esteem and the risk of EDs are associated with dietary knowledge, diet quality, and BMI among medical students at Wroclaw Medical University. We found gender disparities in these associations, highlighting the complex interplay between nutritional behavior, body image, and societal pressures. Among women, higher nutritional knowledge was linked to a tendency toward potentially excessive calorie restriction, reflecting societal pressures for thinness. In contrast, men with greater nutritional knowledge showed lower levels of risk factors for EDs, possibly influenced by expectations of a muscular physique. Overweight individuals tended to prioritize calorie restriction over monitoring food quantity, potentially contributing to weight gain, emphasizing the need for holistic dietary interventions. Better dietary quality was associated with more instances of potentially excessive calorie restriction for both genders, suggesting a complex relationship between dietary habits and restrictive eating practices influenced by societal norms. Lower self-body esteem across both genders was associated with a higher BMI, underscoring the influence of societal pressures on body image. Despite gender differences in nutritional behavior, both genders exhibited similar levels of nutritional knowledge. Women had better dietary quality, while men had higher BMIs, consistent with broader trends.

Future educational initiatives targeting both the general public and medical education programs should prioritize promoting balanced dietary habits and positive body image perceptions, particularly addressing the disproportionate impact of societal pressures on women. Psychotherapeutic interventions show promise in enhancing self-esteem and mitigating symptoms of anxiety and burnout among medical students, suggesting their potential efficacy in preventing and addressing eating disorders. Tailored educational programs should consider the divergent effects of dietary knowledge on women and men, emphasizing the consequences of excessive calorie restriction and the importance of regulating portion sizes. Further research is warranted to explore the underlying mechanisms driving gender differences in dietary habits and body image perceptions, informing targeted intervention strategies.

## Figures and Tables

**Table 1:** Characteristics of study participants.

Variables	Total. *N* = 879 [IQR]. (%)
**Age mean**	19.4 [18.0–20.0]
**Gender**	
Male	219 (24.9)
Female	660 (75.1)
**Place of residence**	
Rural area	199 (22.6)
City < 20,000 *	90 (10.2)
City 20,000–100,000 *	204 (23.2)
City 100,000+ *	386 (43.9)
**BMI**	
Underweight	99 (11.3)
Normal weight	679 (77.3)
Overweight or obese	101 (11.5) (from which 16 (1.82) were obese)

Note: *N* is the number of observations; * number of inhabitants.

**Table 2:** Study variable differences between genders.

	Female, *N* = 660			Male, *N* = 219			Female vs. Male	
	Mean	Median	IQR	Mean	Median	IQR	*p*-Value	Z
**BMI**	21.23	20.8	19.3–22.6	23.02	22.9	21.0–24.3	**<0.0001**	**8.282**
**DK**	12.92	14.0	10.0–16.0	12.62	14.0	10.0–16.0	0.6614	0.438
**DtSc**	10.80	9.36	1.5–19.1	2.97	1.7	−4.26–9.28	**<0.0001**	**8.281**
**DI**	8.19	6.0	3.0–12.0	7.13	5.0	2.0–9.0	0.0892	1.700
**B&FP**	2.37	1.0	0.0–3.0	2.01	0.0	0.0–3.0	0.2653	1.114
**OC**	3.14	2.0	0.0–5.0	3.03	2.0	0.0–4.0	0.9883	0.015
**SA**	48.10	48.0	41.0–55.0					
**WC**	35.72	36.0	30.0–43.0					
**PC**	31.56	31.0	27.0–37.0					
**PA**				41.56	40.0	34.0–50.0		
**UB**				33.02	33.0	27.0–39.0		
**PC**				47.87	48.0	39.0–56.0		

Note: Numbers in bold are statistically significant (*p* < 0.05); DK—Dietary Knowledge; DtSc—Dietary Score; DI—Dieting Scale; B&FP—Bulimia and Food Preoccupation; OC—Oral Control; SA—Sexual Attractiveness; WC—Weight Concern; PC—Physical Condition; PA—Physical Attractiveness; and UB—Upper Body Strength.

**Table 3:** Linear regression analysis of study variables in females.

	Model 1	Model 2
	β (95% CI)	β (95% CI)
**Dieting Scale**		
Dietary Knowledge	**0.0864 (0.0084–0.1643)**	**0.0854 (0.0079–0.1630)**
Dietary Score	**0.0785 (0.0006–0.1565)**	0.0749 (−0.0027–0.1525)
BMI		**0.1052 (0.0296–0.1809)**
R^2^	**0.0167**	**0.0277**
F	**5.5667**	**6.2336**
**Bulimia and Food Preoccupation**		
Dietary Knowledge	0.0113 (−0.0673–0.0899)	0.0110 (−0.0676–0.0896)
Dietary Score	−0.0137 (−0.0923–0.0649)	−0.0150 (−0.0936–0.0636)
BMI		0.0385 (−0.0382–0.1151)
R^2^	0.0002	0.0017
F	0.0808	0.3776
**Oral Control**		
Dietary Knowledge	−0.0074 (−0.0859–0.0712)	−0.0066 (−0.0850–0.0718)
Dietary Score	−0.0223 (−0.1008–0.0563)	−0.0195 (−0.0979–0.0589)
BMI		**−0.0813** (−0.1577–−0.0048)
R^2^	0.0006	0.0072
F	0.2048	1.5904
**Sexual Attractiveness**		
Dietary Knowledge	0.0550 (−0.0234–0.1333)	0.0565 (−0.0209–0.1338)
Dietary Score	0.0422 (−0.0362–0.1206)	0.0478 (−0.0296–0.1252)
BMI		**−0.1652** (−0.2407–−0.0898)
R^2^	0.0058	**0.0331**
F	1.9303	**7.4855**
**Weight Concern**		
Dietary Knowledge	−0.0001 (−0.0786–0.0785)	0.0030 (−0.0712–0.0771)
Dietary Score	0.0302 (−0.0484–0.1088)	0.0415 (−0.0327–0.1157)
BMI		**−0.3333** (−0.4056–−0.2610)
R^2^	0.0009	**0.1118**
F	0.2996	**27.5299**
**Physical Condition**		
Dietary Knowledge	0.0366 (−0.0416–0.1147)	0.0384 (−0.0382–0.1150)
Dietary Score	**0.0933** (0.0151–0.1714)	**0.1001** (0.0235–0.1768)
BMI		**−0.2028** (−0.2775–−0.1281)
R^2^	**0.0116**	**0.0526**
F	**3.8429**	**12.1467**

Note: Numbers in bold are statistically significant (*p* < 0.05).

**Table 4:** Linear regression analysis of study variables in males.

	Model 1	Model 2
	β (95% CI)	β (95% CI)
**Dieting Scale**		
Dietary Knowledge	**−0.2039** (−0.3326–−0.0752)	**−0.2070** (−0.3341–−0.0799)
Dietary Score	**0.2673** (0.1385–0.3960)	**0.2493** (0.1215–0.3771)
BMI		**0.1656** (0.0390–0.2922
R^2^	**0.0974**	**0.1245**
F	**11.6598**	**10.1931**
**Bulimia and Food Preoccupation**		
Dietary Knowledge	**−0.2681** (−0.3987–−0.1375)	**−0.2687** (−0.3995–−0.1379)
Dietary Score	0.0704 (−0.0602–0.2010)	0.0669 (−0.0647–0.1984)
BMI		0.0324 (−0.0979–0.1627)
R^2^	**0.0714**	**0.0725**
F	**8.3096**	**5.6005**
**Oral Control**		
Dietary Knowledge	**−0.2087** (−0.3412–−0.0762)	**−0.2060** (−0.3374–−0.0746)
Dietary Score	0.0603 (−0.0722–0.1928)	0.0761 (−0.0561–0.2082)
BMI		**−0.1453** (−0.2762–−0.0145)
R^2^	**0.0436**	**0.0645**
F	**4.9241**	**4.9382**
**Physical Attractiveness**		
Dietary Knowledge	−0.0164 (−0.1508–0.1179)	−0.0137 (−0.1470–0.1195)
Dietary Score	0.1298 (−0.0046–0.2641)	**0.1457** (0.0117–0.2797)
BMI		**−0.1466** (−0.2793–−0.0139
R^2^	0.0165	**0.0377**
F	1.8118	**2.8092**
**Upper Body Strength**		
Dietary Knowledge	−0.0297 (−0.1633–0.1040)	−0.0294 (−0.1634–0.1045)
Dietary Score	**0.1669** (0.0332–0.3005)	**0.1682** (0.0335–0.3030)
BMI		−0.0128 (−0.1462–0.1206)
R^2^	0.0273	0.0275
F	3.0320	2.0242
**Physical Condition**		
Dietary Knowledge	−0.0698 (−0.2036–0.0639)	−0.0668 (−0.1990–0.0654)
Dietary Score	**0.1560** (0.0223–0.2897)	**0.1739** (0.0410–0.3069)
BMI		**−0.1650** (−0.2967–−0.0334)
R^2^	0.0261	**0.0530**
F	2.8950	**4.0110**

Note: Numbers in bold are statistically significant (*p* < 0.05).

## Data Availability

The datasets used and analyzed during the current study are available from the corresponding author upon reasonable request. The data are not publicly available due to the inclusion of information that could compromise the privacy of the research participants.

## References

[1] Weiselberg E.C., Gonzalez M., Fisher M. (2011). Eating Disorders in the Twenty-First Century. Minerva Ginecol..

[2] Rodgers R.F., Melioli T. (2016). The Relationship Between Body Image Concerns, Eating Disorders and Internet Use, Part I: A Review of Empirical Support. Adolesc. Res. Rev..

[3] Pinhas L., Morris A., Crosby R.D., Katzman D.K. (2011). Incidence and Age-Specific Presentation of Restrictive Eating Disorders in Children: A Canadian Paediatric Surveillance Program Study. Arch. Pediatr. Adolesc. Med..

[4] Benowitz-Fredericks C.A., Garcia K., Massey M., Vasagar B., Borzekowski D.L.G. (2012). Body Image, Eating Disorders, and the Relationship to Adolescent Media Use. Pediatr. Clin. N. Am..

[5] Jovanovski N., Jaeger T. (2022). Demystifying ‘Diet Culture’: Exploring the Meaning of Diet Culture in Online ‘Anti-Diet’ Feminist, Fat Activist, and Health Professional Communities. Women’s Stud. Int. Forum.

[6] Walker D.C., White E.K., Srinivasan V.J. (2018). A Meta-Analysis of the Relationships between Body Checking, Body Image Avoidance, Body Image Dissatisfaction, Mood, and Disordered Eating. Int. J. Eat. Disord..

[7] Fiuza A., Rodgers R.F. (2023). The Effects of Brief Diet and Anti-Diet Social Media Videos on Body Image and Eating Concerns among Young Women. Eat. Behav..

[8] Akbar M. (2022). Effect of Family Pressure, Peer Pressure, and Media Pressure on Body Image Dissatisfaction among Women. J. Bus. Soc. Rev. Emerg. Econ..

[9] Kapoor I., Sharma S., Khosla M. (2020). Social Anxiety Disorder Among Adolescents in Relation to Peer Pressure and Family Environment. Biosci. Biotechnol. Res. Commun..

[10] Azad M.C., Fraser K., Rumana N., Abdullah A.F., Shahana N., Hanly P.J., Turin T.C. (2015). Sleep Disturbances among Medical Students: A Global Perspective. J. Clin. Sleep Med..

[11] Shah M., Hasan S., Malik S., Sreeramareddy C.T. (2010). Perceived Stress, Sources and Severity of Stress among Medical Undergraduates in a Pakistani Medical School. BMC Med. Educ..

[12] Wege N., Muth T., Li J., Angerer P. (2016). Mental Health among Currently Enrolled Medical Students in Germany. Public Health.

[13] Dahlin M.E., Runeson B. (2007). Burnout and Psychiatric Morbidity among Medical Students Entering Clinical Training: A Three Year Prospective Questionnaire and Interview-Based Study. BMC Med. Educ..

[14] Enns M.W., Cox B.J., Sareen J., Freeman P. (2001). Adaptive and Maladaptive Perfectionism in Medical Students: A Longitudinal Investigation. Med. Educ..

[15] Al-Asadi J. (2014). Perceived stress and eating habits among medical students. Int. J. Med. Pharm. Sci. (IJMPS).

[16] Din Z., Iqbal K., Khan I., Abbas M., Ghaffar F., Iqbal Z., Iqbal M., Ilyas M., Suleman M., Iqbal H. (2019). Tendency Towards Eating Disorders and Associated Sex-Specific Risk Factors Among University Students. Noro Psikiyatr. Ars..

[17] Jahrami H., Dewald-Kaufmann J., Faris M.A.-I., AlAnsari A.M.S., Taha M., AlAnsari N. (2020). Prevalence of Sleep Problems among Medical Students: A Systematic Review and Meta-Analysis. J. Public Health.

[18] Cooper A.R., Loeb K.L., McGlinchey E.L. (2020). Sleep and Eating Disorders: Current Research and Future Directions. Curr. Opin. Psychol..

[19] Jahrami H., Sater M., Abdulla A., Faris M.A.-I., AlAnsari A. (2019). Eating Disorders Risk among Medical Students: A Global Systematic Review and Meta-Analysis. Eat Weight Disord..

[20] Qian J., Wu Y., Liu F., Zhu Y., Jin H., Zhang H., Wan Y., Li C., Yu D. (2022). An Update on the Prevalence of Eating Disorders in the General Population: A Systematic Review and Meta-Analysis. Eat Weight Disord..

[21] Regis J.M.O., Ramos-Cerqueira A.T.A., Lima M.C.P., Torres A.R. (2018). Social Anxiety Symptoms and Body Image Dissatisfaction in Medical Students: Prevalence and Correlates. J. Bras. Psiquiatr..

[22] Sugimoto S., Recker D., Halvorson E.E., Skelton J.A. (2023). Are Future Doctors Prepared to Address Patients’ Nutritional Needs? Cooking and Nutritional Knowledge and Habits in Medical Students. Am. J. Lifestyle Med..

[23] Belogianni K., Ooms A., Lykou A., Moir H.J. (2022). Nutrition Knowledge among University Students in the UK: A Cross-Sectional Study. Public Health Nutr..

[24] Coppoolse H.L., Seidell J.C., Dijkstra S.C. (2020). Impact of Nutrition Education on Nutritional Knowledge and Intentions towards Nutritional Counselling in Dutch Medical Students: An Intervention Study. BMJ Open.

[25] Mancin S., Sguanci M., Cattani D., Soekeland F., Axiak G., Mazzoleni B., De Marinis M.G., Piredda M. (2023). Nutritional Knowledge of Nursing Students: A Systematic Literature Review. Nurse Educ. Today.

[26] Marzena J.-Z., Jan G., Lidia W., Jolanta C., Grzegorz G., Anna K.-D., Wojciech R., Agata W., Katarzyna P., Beata S. (2020). KomPAN® Dietary Habits and Nutrition Beliefs Questionnaire for People 15–65 Years Old, Version 1.1.—Interviewer Administered Questionnaire. KomPAN^®^ Dietary Habits and Nutrition Beliefs Questionnaire and the Manual for Developing of Nutritional Data.

[27] Kowalkowska J., Wadolowska L., Czarnocinska J., Czlapka-Matyasik M., Galinski G., Jezewska-Zychowicz M., Bronkowska M., Dlugosz A., Loboda D., Wyka J. (2018). Reproducibility of a Questionnaire for Dietary Habits, Lifestyle and Nutrition Knowledge Assessment (KomPAN) in Polish Adolescents and Adults. Nutrients.

[28] Kosendiak A.A., Adamczak B.B., Kuźnik Z., Makles S. (2024). Impact of Medical School on the Relationship between Nutritional Knowledge and Sleep Quality—A Longitudinal Study of Students at Wroclaw Medical University in Poland. Nutrients.

[29] Franzoi S.L., Shields S.A. (1984). The Body Esteem Scale: Multidimensional Structure and Sex Differences in a College Population. J. Personal. Assess..

[30] Lipowska M., Lipowski M. (2014). Polish Normalization of the Body Esteem Scale. Health Psychol. Rep..

[31] Garner D.M., Olmsted M.P., Bohr Y., Garfinkel P.E. (1982). The Eating Attitudes Test: Psychometric Features and Clinical Correlates. Psychol. Med..

[32] Rogoza R., Brytek-Matera A., Garner D. (2016). Analysis of the EAT-26 in a Non-Clinical Sample. Arch. Psych. Psych..

[33] Włodarczyk-Bisaga K., Dolan B. (1996). A Two-Stage Epidemiological Study of Abnormal Eating Attitudes and Their Prospective Risk Factors in Polish Schoolgirls. Psychol. Med..

[34] Faw M.H., Davidson K., Hogan L., Thomas K. (2021). Corumination, Diet Culture, Intuitive Eating, and Body Dissatisfaction among Young Adult Women. Pers. Relatsh..

[35] Voges M.M., Giabbiconi C.-M., Schöne B., Waldorf M., Hartmann A.S., Vocks S. (2019). Gender Differences in Body Evaluation: Do Men Show More Self-Serving Double Standards Than Women?. Front. Psychol..

[36] Ribeiro A.S., Nunes J.P., Schoenfeld B.J., Aguiar A.F., Cyrino E.S. (2019). Effects of Different Dietary Energy Intake Following Resistance Training on Muscle Mass and Body Fat in Bodybuilders: A Pilot Study. J. Hum. Kinet..

[37] Magallares A. (2016). Drive for Thinness and Pursuit of Muscularity: The Role of Gender Ideologies. Univ. Psychol..

[38] O’Shea L. (2020). Diet Culture and Instagram: A Feminist Exploration of Perceptions and Experiences Among Young Women in the Midwest of Ireland. Grad. J. Gend. Glob. Right.

[39] Most J., Redman L.M. (2020). Impact of Calorie Restriction on Energy Metabolism in Humans. Exp. Gerontol..

[40] Pollert G.A., Engel S.G., Schreiber-Gregory D.N., Crosby R.D., Cao L., Wonderlich S.A., Tanofsky-Kraff M., Mitchell J.E. (2013). The Role of Eating and Emotion in Binge Eating Disorder and Loss of Control Eating. Int. J. Eat. Disord..

[41] Stice E., Spoor S., Bohon C., Veldhuizen M., Small D. (2008). Relation of Reward from Food Intake and Anticipated Food Intake to Obesity: A Functional Magnetic Resonance Imaging Study. J. Abnorm. Psychol..

[42] He J., Cai Z., Fan X. (2017). Prevalence of binge and loss of control eating among children and adolescents with overweight and obesity: An exploratory meta-analysis. Int. J. Eat. Disord..

[43] Farina E.K., Thompson L.A., Knapik J.J., Pasiakos S.M., Lieberman H.R., Mcclung J.P. (2020). Diet Quality Is Associated with Physical Performance and Special Forces Selection. Med. Sci. Sports Exerc..

[44] Pop C. (2016). Self-Esteem and Body Image Perception in a Sample of University Students. Eurasian J. Educ. Res..

[45] Sylvia Z., King T.K., Morse B.J. (2014). Virtual Ideals: The Effect of Video Game Play on Male Body Image. Comput. Hum. Behav..

[46] İpek Kalender G. The Portrayal of Ideal Beauty Both in the Media and in the Fashion Industry and How These Together Lead to Harmful Consequences Such as Eating Disorders. Proceedings of the 10th International Conference on Humanities, Psychology and Social Sciences.

[47] Jarman H.K., Marques M.D., McLean S.A., Slater A., Paxton S.J. (2021). Social Media, Body Satisfaction and Well-Being among Adolescents: A Mediation Model of Appearance-Ideal Internalization and Comparison. Body Image.

[48] Uchôa F.N.M., Uchôa N.M., Daniele T.M.d.C., Lustosa R.P., Garrido N.D., Deana N.F., Aranha Á.C.M., Alves N. (2019). Influence of the Mass Media and Body Dissatisfaction on the Risk in Adolescents of Developing Eating Disorders. Int. J. Environ. Res. Public Health.

[49] Lopes V.P., Malina R.M., Gomez-Campos R., Cossio-Bolaños M., Arruda M.d., Hobold E. (2019). Body Mass Index and Physical Fitness in Brazilian Adolescents. J. Pediatr..

[50] Gutin I. (2018). In BMI We Trust: Reframing the Body Mass Index as a Measure of Health. Soc. Theory Health.

[51] Bator E., Habanova M., Broniecka A., Wyka J., Bronkowska M. Porównanie Poziomu Wiedzy Żywieniowej Studentów Polskich i Słowackich w Zakresie Źródeł Pokarmowych Wybranych Składników Odżywczych. https://www.ptfarm.pl/wydawnictwa/czasopisma/bromatologia-i-chemia-toksykologiczna/117/-/15714.

[52] Kosendiak A.A., Wysocki M., Krysiński P., Kuźnik Z., Adamczak B. (2023). Impact of the COVID-19 Pandemic on Physical Activity, Smoking, Alcohol Use, and Mental Well-Being—A Longitudinal Study of Nursing Students at Wroclaw Medical University in Poland. Front. Public Health.

[53] Sidor A., Rzymski P. (2020). Dietary Choices and Habits during COVID-19 Lockdown: Experience from Poland. Nutrients.

[54] Sumalla-Cano S., Forbes-Hernández T., Aparicio-Obregón S., Crespo J., Eléxpuru-Zabaleta M., Gracia-Villar M., Giampieri F., Elío I. (2022). Changes in the Lifestyle of the Spanish University Population during Confinement for COVID-19. Int. J. Environ. Res. Public Health.

[55] Adamczak B., Kuźnik Z., Makles S., Kosendiak A. (2023). Physical activity of ukrainian and polish medical students in the beginning of the war in Ukraine. Health Probl. Civiliz..

[56] Alotaibi A.D., Alosaimi F.M., Alajlan A.A., Bin Abdulrahman K.A. (2020). The Relationship between Sleep Quality, Stress, and Academic Performance among Medical Students. J. Fam. Community Med..

[57] Ho A.S.L., Soh N.L., Walter G., Touyz S. (2011). Comparison of Nutrition Knowledge among Health Professionals, Patients with Eating Disorders and the General Population. Nutr. Diet..

[58] Pinkhasov R.M., Wong J., Kashanian J., Lee M., Samadi D.B., Pinkhasov M.M., Shabsigh R. (2010). Are Men Shortchanged on Health? Perspective on Health Care Utilization and Health Risk Behavior in Men and Women in the United States. Int. J. Clin. Pract..

[59] Kolarzyk E., Shpakou A., Kleszczewska E., Klimackaya L., Laskiene S. (2012). Nutritional Status and Food Choices among First Year Medical Students. Cent. Eur. J. Med..

[60] Imamura F., Micha R., Khatibzadeh S., Fahimi S., Shi P., Powles J., Mozaffarian D. (2015). Dietary Quality among Men and Women in 187 Countries in 1990 and 2010: A Systematic Assessment. Lancet Glob. Health.

[61] Linardon J., Kothe E.J., Fuller-Tyszkiewicz M. (2019). Efficacy of Psychotherapy for Bulimia Nervosa and Binge-Eating Disorder on Self-Esteem Improvement: Meta-Analysis. Eur. Eat. Disord. Rev..

[62] Regehr C., Glancy D., Pitts A., LeBlanc V.R. (2014). Interventions to Reduce the Consequences of Stress in Physicians: A Review and Meta-Analysis. J. Nerv. Ment. Dis..

